# Biocidal polymers: synthesis and antimicrobial properties of benzaldehyde derivatives immobilized onto amine-terminated polyacrylonitrile

**DOI:** 10.1186/1752-153X-6-111

**Published:** 2012-10-01

**Authors:** Abdullah Alamri, Mohamed H El-Newehy, Salem S Al-Deyab

**Affiliations:** 1 Petrochemical Research Chair, Department of Chemistry, College of Science, King Saud University, P.O. Box: 2455, Riyadh, 11451, Saudi Arabia; 2 Department of Chemistry, Faculty of Science, Tanta University, Tanta, 31527, Egypt

## Abstract

**Background:**

The design and applications of antimicrobial polymers is a growing field. Antimicrobial polymers can help to solve the problems associated with the use of conventional antimicrobial agents. Polymers with active functional groups can act as a carrier system for antimicrobial agents. In our study, we aim to prepare and develop some antimicrobial polymers for biomedical applications and water treatment.

**Results:**

The antimicrobial polymers based on polyacrylonitrile (PAN) were prepared. Functional groups were created onto polyacrylonitrile via amination using different types of diamines such as ethylenediamine (EDA) and hexamethylenediamine (HMDA) to yield amine-terminated polymers. Antimicrobial polymers were obtained by immobilization of benzaldehyde and its derivatives which include, 4-hydroxybenzaldehyde and 2,4-dihydroxybenzaldehyde onto amine-terminated polymers. The antimicrobial activity of the prepared polymers against different types of microorganisms including Gram-positive bacteria (*Staphylococcus aureus),* Gram-negative bacteria (*Pseudomonas aeruginosa*; *Escherichia coli*; and *Salmonella typhi*) as well as fungi (*Aspergillus flavus*, *Aspergillus niger*, *Candida albicans*, *Cryptpcoccus neoformans*) were explored by the cut plug method and viable cell counting methods.

**Conclusions:**

Amine-terminated polyacrylonitrile were used as a novel polymeric carrier for benzaldehyde derivatives as antimicrobial agents. The prepared polymers can inhibit the growth of the microorganisms. The activity was varied according to the tested microorganism as well as the polymer microstructure. It was found that the activity increased with increasing the number phenolic hydroxyl group of the bioactive group. Finally, it is anticipated that the prepared antimicrobial polymers would be of great help in the field of biomedical applications and biological water treatment.

## Background

Biocidal polymer is a polymer that has the ability to kill microorganisms, by acting as a source of sterilizing ions or molecules [1]. Generally, the use of conventional antimicrobial agents is associated with the problems of residual toxicity of these agents which can cause more serious problems to the environment. For example, in the case of using these antimicrobial agents in food packaging, there is a risk of diffusion of these agents into the food causing various problems [2,3]. In water treatment, the most popular treatment method to disinfect and sterilize water is to use chlorine and other related chemicals. However, their residues can become concentrated in the food chain and in the environment as well as the possible formation of halomethane analogues that are suspected of being carcinogenic should lead to the avoidance of their use [2]. Due to the associated problems result from the use of conventional antimicrobial agents; the idea of polymeric antimicrobial agents appeared to be an attractive alternative. Nowadays, there is an increasing interest in selective antimicrobial polymers whose potency against bacteria and non-toxicity towards mammalian cells distinguishes them from most polymeric biocides that are broadly poisonous [4-10]. In addition, the use of polymeric antimicrobial agents have the advantages that they are nonvolatile, chemically stable, and find it difficult to permeate through the skin of man or animal and may enhance the efficacy of some existing antimicrobial agents and minimize the environmental problems accompanying the residual toxicity of the agents in addition to prolonging their lifetime [2,11-14]. Therefore, the use of polymeric materials with antimicrobial properties gains an increasing interest from both academic and industrial point of view.

Polymers can act as matrix for the materials holding the antimicrobial agents [15]. Phenols are one of these antimicrobial agents that act in the bacteria membrane. They interact with the surface of the cell and lead to cell death through disintegration of the cell membrane and release of intracellular constituents. Phenols also cause intracellular coagulation of cytoplasmic constituents leading to cell death or inhibition of cell growth [7,16]. Benzaldehyde derivatives analogue of phenols are widely used as environmentally safe antimicrobial compounds. Considering its broad spectrum inhibitory activities, they are employed as bactericide, fungicide and algaecide [17].

Moreover, functional polymers have the potential advantages of small molecules with the same functional groups. Their usefulness is related to both the functional groups and to their polymeric nature whose characteristic properties depend mainly on the extraordinarily large size of the molecules [18,19]. Polyacrylonitrile (PAN) molecular chains carry a cyano group, which can be modified. It is hydrolysable and can be adjusted to achieve functionality in a number of applications [20-24]. In our approach, functional groups were created onto polyacrylonitrile via amination using different types of diamines to yield amine-terminated polymers as polymeric carriers for antimicrobial agents such as benzaldehyde derivatives. The chemical structure of the prepared polymers was confirmed by FTIR Spectra, and TGA. The antimicrobial activity of the prepared polymers against different types of microorganisms including Gram-positive bacteria (*Staphylococcus aureus),* Gram-negative bacteria (*Pseudomonas aeruginosa*; *Escherichia coli*; and *Salmonella typhi*) as well as fungi (*Aspergillus flavus*, *Aspergillus niger*, *Candida albicans*, *Cryptpcoccus neoformans*) were explored by the cut plug method and viable cell counting methods. The prepared biocidal polymers are water-insoluble; therefore, they can be used safely in sterilizing drinking water and many other applications, such as disinfecting water supplies, swimming pools, hot-tubs, industrial water systems, and other applications where a sanitized water supply is required.

## Experimental

### Materials

Acrylonitrile (AN) was purchased from Loba Chemie, Spain. Ethylenediamine (EDA), and hexamethylenediamine (HMDA) were purchased from Across. Benzaldehyde (Bz), 4-hydroxybenzaldehyde (4-HO-Bz) and 2,4-dihydroxybenzaldehyde (2,4-Di-HO-Bz) were purchased from Across. Pipridine was purchased from BDH Chemicals Ltd Poole England. All solvents were used as received without further purification.

### Characterization techniques

Thermal properties were examined using thermogravimetric analysis (TGA) which was carried on TA-Q500 System of TA; samples of 5–10 mg were heated in the temperature range 30–800°C at a scanning rate of 10°C·min^-1^ under nitrogen atmosphere. Fourier-transformer infrared (FT-IR) Spectra were recorded using TENSOR 27, Bruker.

Full-stained ultra-thin sections were examined using the transmission electron microscope (JEOL-JEM-100SX, Japan) with beam current = 60 μA, and high voltage of 80 kV.

### Polymer synthesis and modification

**Scheme 1:**
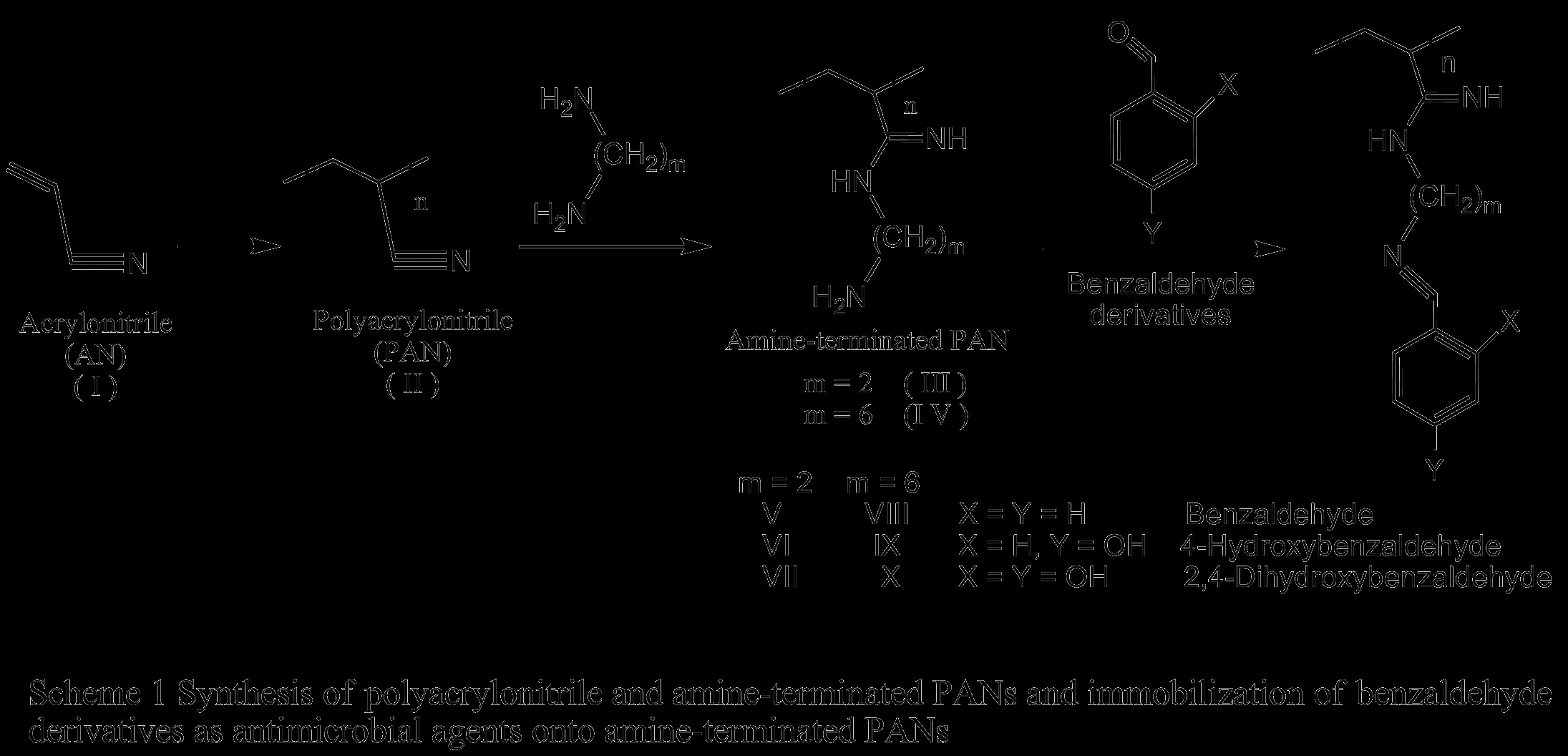
Synthesis of polyacrylonitrile and amine-terminated PANs and immobilization of benzaldehyde derivatives as antimicrobial agents onto amine-terminated PANs.

Polyacrylonitrile (PAN) (II) was prepared in aqueous solution (precipitation polymerization) according to a previously reported procedure [25,26]. PAN (II) was prepared with a redox system in aqueous solution at room temperature with stirring under nitrogen atmosphere. Then, sodium disulfite solution and ferrous sulfate solution were added followed by addition of potassium peroxodisulfate solution. The precipitated polymer (II) was filtered off, washed with distilled water and methanol, respectively (Scheme 1). 

Amine-terminated polymers (III & IV) were prepared according to the procedure described by El-Newehy *et al.*[26] by the reaction of polyacrylonitrile with ethylenediamine (EDA), and hexamethylenediamine (HMDA). In brief: In a 100 mL round-bottomed flask, it was placed an excess amount of the diamine (10-fold) in absolute ethanol, then PAN (II) was added portion-wise over 1 h with stirring under nitrogen. The reaction mixture was stirred at room temperature for 1–2 h and at 70°C for 12 h. The product was filtered, washed with methanol (Scheme 1).

### Immobilization of benzaldehyde derivatives as antimicrobial agents onto amine-terminated PANs (V - X)

#### General procedure

Benzaldehyde derivatives were immobilized onto amine-terminated polymers (III & IV) according to the procedure described previously [27]. In summary; In a 50 mL round-bottomed flask, to a mixture of benzaldehyde derivative and amine-terminated polymers (III & IV) (2.5 g) in absolute ethanol (30 mL), two drops of piperidine was added (Table 1). The mixture was stirred at room temperature for 48 h and at 90°C for 72 h with stirring. The product was recovered by filtration, washed with alcohol and then was dried in oven under vacuum at 40°C for 48 h to give a yellowish amorphous product (Scheme 1). 

**Table 1:** Summary of the used quantity of benzaldehyde derivatives and yield (%)

**Polymer Code**	**g(mmol)**					**Yield (%)**
	**III**	**IV**	**Bz^1^**	**4-HO-Bz^2^**	**2,4-Di-HO-Bz^3^**	
V	2.5(22.1)	-	5(47.2)	-	-	80
VI			-	5(41.0)	-	72
VII			-	-	5(36.2)	81
VIII	-	2.5(14.8)	5(47.2)	-	-	73
IX			-	5(41.0)	-	74
X			-		5(36.2)	90

^1^Benzaldehyde; ^2^4-Hydroxybezaldehyde; ^3^2,4-Dihydroxybenzaldehyde.

### Antimicrobial assessments

Antimicrobial assessments were carried out at Microbiology Unit, Department of Botany, Faculty of Science, Tanta University, Tanta, Egypt.

### Tested microorganisms and biological tests

The microorganisms include the Gram-negative bacteria *Escherichia coli*, *Pseudomonas aeruginosa*, and *Salmonella typhi,* the Gram-positive bacteria *Staphylococcus aureus*, and the filamentous fungi *Aspergillus niger, Aspergillus flavus*, yeast form fungi *Candida albicans,* and *Cryptpcoccus neoformans*.

Bacteria were maintained on nutrient agar, and the fungi were kept on Sabouroud's agar slopes. All fungi were isolated from patients of Tanta University hospitals, and were authenticated by Assuit Mycology Center, Faculty of Science, Assuit University, Assuit, Egypt. All bacteria were isolated from patients of Tanta University hospitals, and were authenticated by Bacteriology Research Unit, Faculty of Science, Al-Azhar University, Cairo, Egypt.

### Screening of antimicrobial activity for tested polymers using cut plug method

Cut plug method recorded by Pridham, *et.al*. [28] was employed to determine the antimicrobial activity of the prepared polymers as following: Freshly prepared spore suspension of different tested microorganisms (0.5 mL of about 10^6^ cells/mL) was mixed with 9.5 mL of melting sterile Sabouraud's dextrose medium (for fungi) or nutrient agar medium (for bacteria) at 45°C, poured on sterile Petri dishes, and left to solidify at room temperature. Regular wells were made in the inoculated agar plates by a sterile cork borer of 0.7 mm diameter. Each well was filled with 20 mg of each tested powder. Three replicates were made for each test, and all plates were incubated at 27°C for 72 h for fungi, and at 32°C for 24 h for bacteria. Then the average diameters of inhibition zones were recorded in millimeters (mm), and compared for all plates.

### MIC determination for the most efficient polymers against tested microorganisms

Half-fold serial dilutions were made for selected polymers in order to prepare concentrations of 6.25, 12.5, 25, 50 and 100 mg/mL in distilled water, zero concentration was considered as a negative control. A previously prepared pure spore suspension of each tested microorganism (0.5 mL of about 10^6^ cells/mL) was mixed with 9.5 mL of each concentration in sterile test tubes, incubated at 27°C for 72 h for fungi, and at 32°C for 24 h for bacteria, then optical density of growth was measured by spectrophotometer (Optima SP-300, Japan) at 620 nm for each incubated mixture, results were represented graphically, and MIC was recorded for each tested material [29].

### Methodology for transmission electron microscope (TEM)

TEM analyses were carried out in Scanning Electron Microscope Unit, Histology Department, Faculty of Medicine, Tanta University, Tanta 31527, Egypt.

For studying the effect of the antimicrobial agents on the ultra-structure of microbial cells, tested microorganism was cultured on the appropriate liquid nutrition medium to get a cell suspension of 5x10^6^ cells/mL, and then mixed with the selected polymer of the previously recorded MIC. The mixture was incubated overnight on a shaking incubator of 60 rpm at the appropriate temperature. Then the treated mixture was centrifuged at 3000 rpm for 20 min, washed with sterile saline solution, and re-centrifuged to collect the cell pellet in a clean Eppendorf tube [30].

Collected cell pellet was fixed by adding 1 mL of 2.5% glutaraldehyde that was buffered in 0.1 M phosphate buffer saline (PBS) of pH = 7.4 (for fixation of cellular protein content, and to stop all culture reactions), and was cooled at 4°C for 2 h. Fixed sample was washed with 1% osmic acid for 30 min (for fixation of lipid cell content), washed 3 times with PBS (10 min. for each time), and dehydrated in ascending ethanol concentrations (30, 50, 70, 90, and absolute alcohol) for 30 min for each concentration, then dehydrated sample was infiltrated with acetone for 1 h.

For TEM analysis, sample was embedded in araldite 502 resin to build a plastic mold (for complete fixation of all cell contents), that were cut into semi-thin sections in the ultra-cut microtome (LEICA ultracut UCT, Japan), stained with 1% Toluidine blue, examined to confirm the success of sample preparation, then ultra-thin sections were prepared, stained with uranyl acetate, and counter stained with lead citrate [31].

Full-stained ultrathin sections were examined, and photographed under the appropriate magnification (at 10000X for larger cells of fungi, or 20000X for smaller cells of bacteria), using the transmission electron microscope (JEOL-JEM-100SX, Japan) with beam current = 60 μA, and high voltage of 80 kV.

### Physiological measurements

Physiological tests were carried out in Microbial physiology lab. For fungal and bacterial measurements, Microbiology Department, Faculty of Science, Al-Azhar University, Cairo, Egypt.

For studying the effect of the antimicrobial agents on the physiological parameters of microbial cells, tested microorganism was cultured on the appropriate liquid nutrition medium to get a cell suspension of 5x10^6^ cells/mL, and then mixed with the tested polymer of the previously recorded MIC. All the mixture was incubated overnight on a shaking incubator of 60 rpm at the appropriate temperature. Then the treated mixture was centrifuged at 3000 rpm for 20 min, washed with sterile distilled water, re-centrifuged to collect the cell pellet in a clean Eppendorf tube; cells were broken for extraction of intracellular content by grinding with glass beads in 1 mL distilled H_2_O, filtered, and supernatants were collected, and stored at 4°C for further analysis.

### Estimation of total soluble cell proteins concentration

Comassie brilliant blue G-250 dye of 100 mg was dissolved in 50 mL of a mixture of 95% ethanol and 100 mL of 85% phosphoric acid, diluted to 1 L with distilled water, and then filtered. After that, 0.1 mL of previously treated cell extract was mixed with 5 mL of Comassie dye and was shacked well for 5 min, the light absorbance of mixture was recorded at λ = 595 nm. Records of absorbance indicate the concentration of the total soluble cell proteins in tested supernatants using a previously drawn standard curve of light absorbance of known concentration of bovine serum albumin as a standard protein stained and was measured in the same way as the unknown sample [32].

### Estimation of total cell ionic content

All minerals and ionic components of the treated cells which were released in the previously treated, and extract of broken cells were stored. Each tested suspension was diluted to 10 mL with distilled water, then the electrode of EC-meter (Sensorex CS200TC lab conductivity sensor, USA) was immersed in the suspension, and the potential of electric current was recorded. The concentration of the total ions present in the tested suspension was indicated by the records of electric current potential, which can be read from a previously prepared standard curve of electric conductivity of known concentration of known electrolyte [33].

## Results and discussion

Benzaldehyde derivatives are widely used as environmentally safe antimicrobial compounds. Considering its broad spectrum inhibitory effect, they are employed as bactericide, fungicide and algaecide. Hydroxybenzaldehydes resemble phenols in biocidal activity against bacteria. They interact with the surface of the cell and lead to cell death through disintegration of the cell membrane and release of intracellular constituents. Phenols and/or hydroxybenzaldehydes also cause intracellular coagulation of cytoplasmic constituents leading to cell death or inhibition of cell growth [7,16]. In our work, we report the use of amine-terminated polymers as novel polymeric carriers for benzaldehyde derivatives as antimicrobial agents (Scheme 1).

### FT-IR spectra

**Figure 1:**
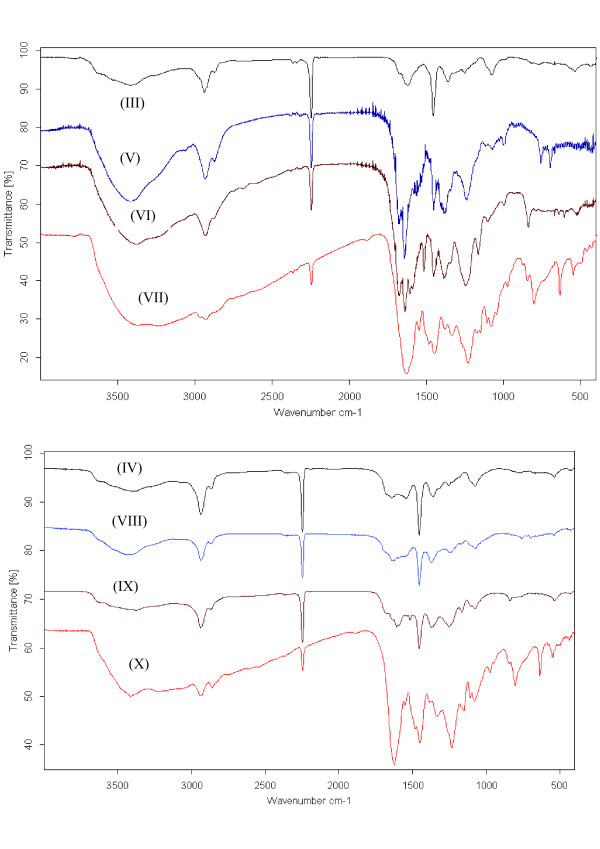
FT-IR Spectra of polymers (III-X).

FTIR spectroscopy was used to prove the immobilization of the antimicrobial agents onto amine-terminated PANs (V-X) as shown in (Table 2) and (Figure 1). Characteristic absorption peaks were observed at 1361–1454 cm^−1^ due to -NH stretching and strong peaks at 2940 cm^−1^ corresponds to -C-H stretching vibration and at 3555 cm^−1^ due to = NH stretching. In addition, the spectra of the antimicrobial polymers (V-X) shows many significant changes; a new band was appeared around 1583 cm^−1^ due to aromatic ring and new band at 3375 and 3414 cm^-1^ which is attributed to the –OH stretching vibration, which in conclusion confirm the immobilization of benzaldehyde onto amine-terminated PANs.

**Table 2:** FTIR absorption frequencies of functional groups in amine-terminated PANs and their antimicrobial polymers (II-X)

**Functional groups**	**Wavenumber (cm^-1^)**								
	**II**	**III**	**IV**	**V**	**VI**	**VII**	**VIII**	**IX**	**X**
-CN stretching	2243	-	-	-	-	-	-	-	-
-C-H stretching	2940	2940	2940	2935	2935	2932	2938	2938	2938
–NH_2_ stretching	-	3415	3339	-	-	-	-	-	-
N–H stretching	-	1545	1561	1552	1531	1546	1453	1453	1450
Aromatic C-H bending		-		757	838	801	810	838	804
-OH stretching	-	-		-	3382	3239	-	3375	3414
=NH stretching	-	3415	3339	3422	3382	3239	3350	3375	3414
-C = N- stretching	-	-	-	1639	1638	1626	1606	1606	1621

### Thermal analysis

**Figure 2:**
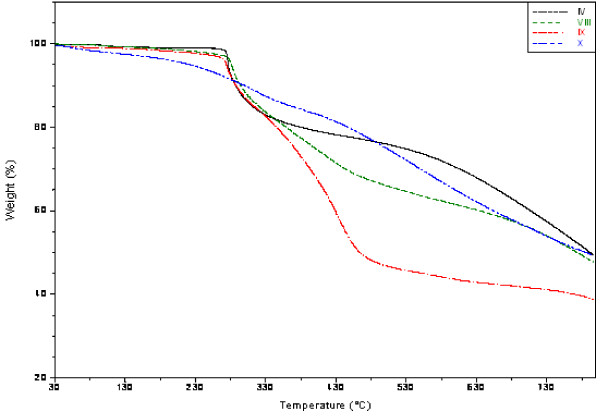
TGA thermograms of polymers (V-X).

The thermogram showed three steps for the degradation of the prepared polymers (II-X) which were performed with a heating rate of 10°C min^-1^ under nitrogen atmosphere as shown in (Figure 2) and the data were summarized in (Table 3).

**Table 3:** Proximate analysis (wt%) of polymers (II-X) based on thermogravimetric analysis

**Polymer code**	**Distribution of volatile ranges (Temperature range)(°C)**			**Residue (%) At 800°C**	***T*_*on*_ (°C)**	**50% Loss at (°C)**
	**Moisture**	**Grafted functional group**	**Remainder PAN**			
	**30-150**	**150-350**	**>350**			
II	0.1	36.6	21.2	36	287	425
III^*^	1.5	14.6	34.6	49	272	790
IV^#^	0.7	20.0	29.8	49	274	791
V	1.4	16.3	37.3	45	296	754
VI	0.1	14.8	37.6	47	294	775
VII	4.6	21.9	18.0	50	388	796
VIII	0.8	18.0	28.8	47	296	773
IX	0.7	19.7	38.5	38	289	464
X	2.5	12.8	34.9	49	342	786

^*^Grafted functional group degradation (150-335°C);^*#*^*Grafted functional group degradation (150-400°C).*

Generally, the first step ranges between room temperature and 150°C for all polymers which may be attributed to the evolution of moisture. The second step of weight loss starts at about 150°C and continues up to 335–400°C due to the degradation of the grafted functional groups. The last step over 350°C up to 800°C which may be due to the degradation of the remainder of polyacrylonitrile chains.

Table 3 summarizes the TGA results of polymers (II-X). The thermal degradation of PAN (II) and amine-terminated PANs (III & IV) was explained in details in our previous work [26]. For the antimicrobial polymers (V-X), the thermogram showed that the onset decomposition temperature was found to be in the range 289-296°C except for polymers (VII and X) were found to be 388 and 342°C, respectively, which may attributed to the forming of hydrogen bond as the number of hydroxyl group increased in the immobilized antimicrobial agent. Moreover, the prepared polymers leave residue around 38–50%.

The obtained data in this study demonstrated a slightly differences in the thermal stabilities between the starting material, PAN and the amine-terminated PANs and an increase in the thermal stability of the antimicrobial polymers (V-X) compared to the starting materials, PAN (II) and the amine-terminated PANs (III & IV) as shown in (Table 3).

### Antimicrobial assessment of polymers (V-X)

#### Effect of spacer and antimicrobial agent microstructure

**Figure 3:**
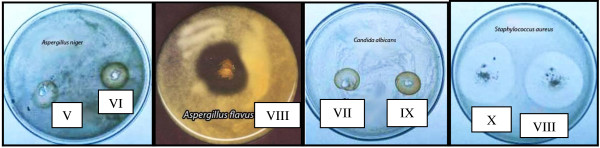
**Inhibition Zones of polymers (V-X) against pathogenic fungi; *Aspergillus niger, Aspergillus flavus, *and *Candida albicans, *against Gram-positive bacteria *Staphylococcus aureus.***

The antimicrobial activity of polymers (V-X) containing phenolic compounds against Gram-negative and positive bacteria as well as fungi were explored by the cut plug method and viable cell counting methods [28]. The obtained results relieved the capability of polymers (V-X) to inhibit the growth of microorganisms as shown in (Table 4) & (Figure 3). Generally, it was found that the diameter of the inhibition zone varied according to the tested microorganism as well as the polymer microstructure. The inhibition zone diameter increased from polymer (V) passed to polymer (VII) due to the increase in the number of phenolic hydroxyl group of the bioactive group. The same conclusion was observed for polymers (VIII-X). At the meantime, the inhibition zone diameter increased from polymer (V) to (VIII), (VI) to (IX) and (VII) to (X) due to the increase in space length between the bioactive group and polymer backbone. Generally, all polymers showed an increase in the inhibitory action against all the tested microorganisms in comparison with their control (III &IV). Also, it was observed that the lowest activity was recorded for polymer (V) against *Escherichia coli* (14 mm), and the highest was recorded for polymer (X) against *Pseudomonas aeruginosa* and *Salmonella typhi* (32 mm). 

**Table 4:** Inhibition zone diameters (mm) produced by 20 mg of tested polymers (V-X) against different bacteria which were maintained on nutrient agar after 24 h and fungi which were kept on Sabouroud's agar slopes after 72 h by the Cut Plug Method on nutrient agar at 30°C

**Tested microorganism**	**Inhibition zone diameter (mm)**							
	***Control***		***Biocidal polymers***					
	**III**	**IV**	**V**	**VI**	**VII**	**VIII**	**IX**	**X**
*Escherichia coli*	10	8	14	19	25	30	23	28
*Pseudomonas aeruginosa*	8	8	15	23	28	30	24	32
*Salmonella typhi*	9	9	16	19	28	32	23	32
*Staphylococcus aureus*	8	10	16	21	27	29	25	30
*Aspergillus niger*	9	10	18	20	26	30	22	28
*Aspergillus flavus*	9	8	15	20	27	31	22	29
*Candida albicans*	10	9	17	19	25	28	22	27
*Cryptpcoccus neoformans*	10	9	17	19	25	29	24	27

### MIC determination for the most efficient polymers against tested microorganisms

**Figure 4:**
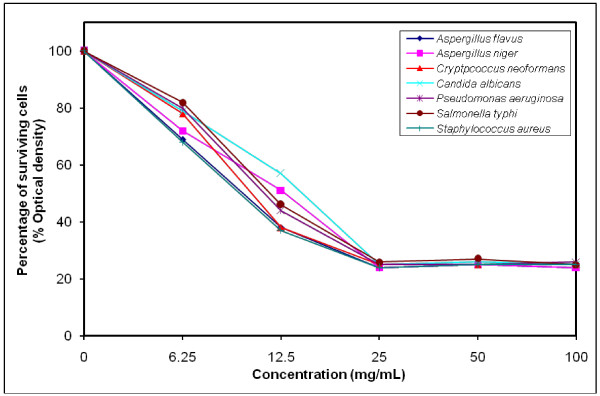
Growth inhibition of different concentration of polymer (V) against different tested microorganisms.

**Figure 5:**
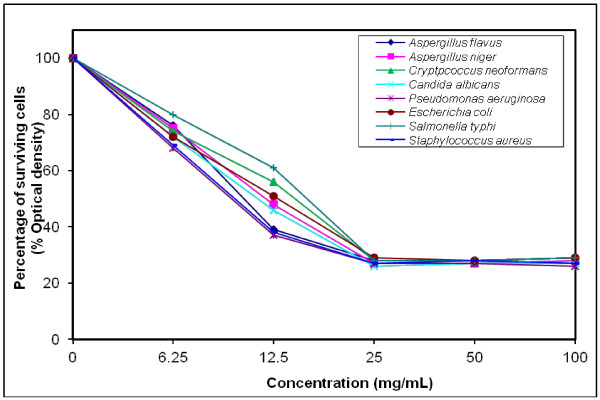
Growth inhibition of different concentration of polymer (VI) against different tested microorganisms.

**Figure 6:**
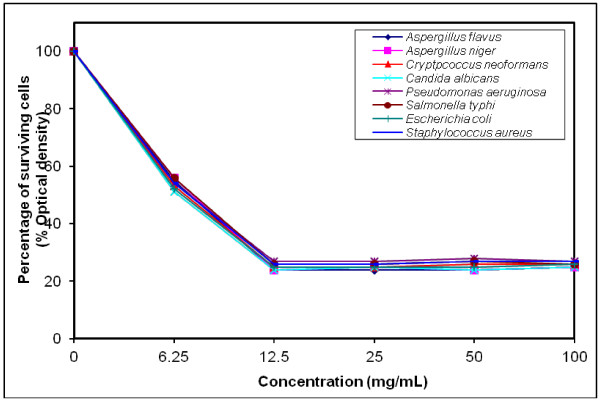
Growth inhibition of different concentration of polymer (VII) against different tested microorganisms.

**Figure 7:**
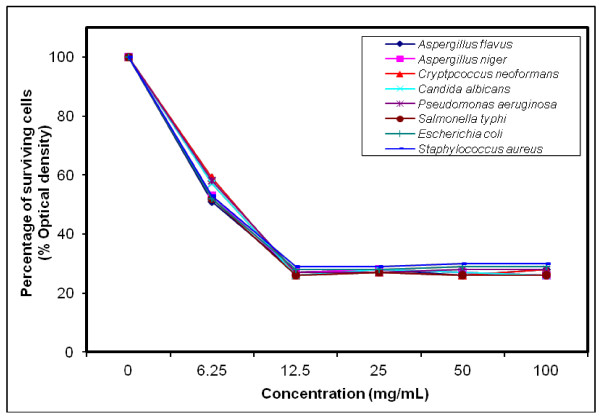
Growth inhibition of different concentration of polymer (VIII) against different tested microorganisms.

**Figure 8:**
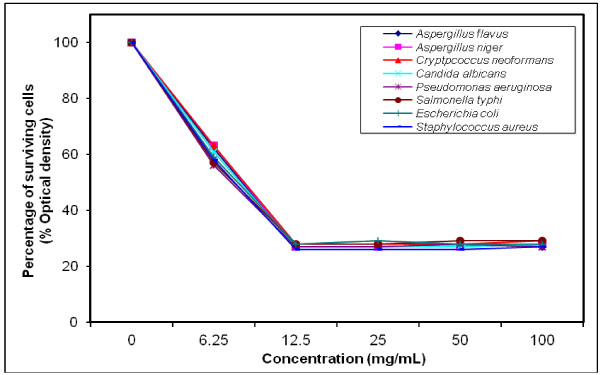
Growth inhibition of different concentration of polymer (IX) against different tested microorganisms.

**Figure 9:**
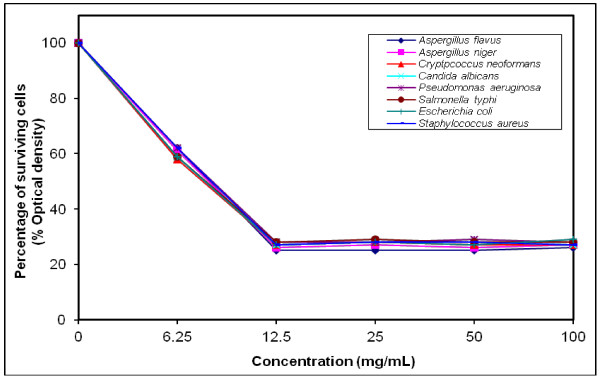
Growth inhibition of different concentration of polymer (X) against different tested microorganisms.

The growth-inhibiting effect was quantitatively determined by percentage of the surviving cells (% Optical density) as shown in (Figures 4, 5, 6, 7, 8, 9). The MIC values for these polymers were determined by using the broth dilution method. We considered zero concentration as a negative control. The polymer concentrations ranged from 6.25-100 mg/mL which were obtained by half-fold serial dilutions. Each solution in the series was mixed with 10^6^ cells/mL of each tested microorganism [29]. 

MIC was recorded at 25 mg/mL for polymers (V & VI), and decreased to 12.5 mg/mL for polymers (VII–X), that possessed higher inhibitory effect on tested microbes due to the increase in space length between the bioactive groups, and the polymer backbone resulting in more released of the inhibitory groups.

Generally, the polymer samples killed 70–75% at MIC concentration the inhibitory effect which was varied according to the polymer microstructure, antimicrobial agent as well as the type of tested microorganisms as shown in (Table 5).

**Table 5:** MIC of selected polymers against different sensitive microorganisms

**Tested microorganism**	**Percentage of surviving cells(% Optical density)**					
	***Biocidal polymers***					
	**V^a^**	**VI^a^**	**VII^b^**	**VIII^b^**	**IX^b^**	**X^b^**
*Escherichia coli*	-	29	25	28	28	27
*Pseudomonas aeruginosa*	25	27	27	27	27	28
*Salmonella typhi*	26	28	26	26	28	28
*Staphylococcus aureus*	24	27	26	29	26	27
*Aspergillus niger*	24	27	24	27	27	26
*Aspergillus flavus*	24	28	24	27	27	25
*Candida albicans*	25	26	24	26	27	27
*Cryptpcoccus neoformans*	25	28	25	26	28	27

^a^ MIC = 25 mg/mL. ^b^ MIC = 12.5 mg/mL.

### Transmission electron micrograph (TEM) of the microbes

**Figure 10:**
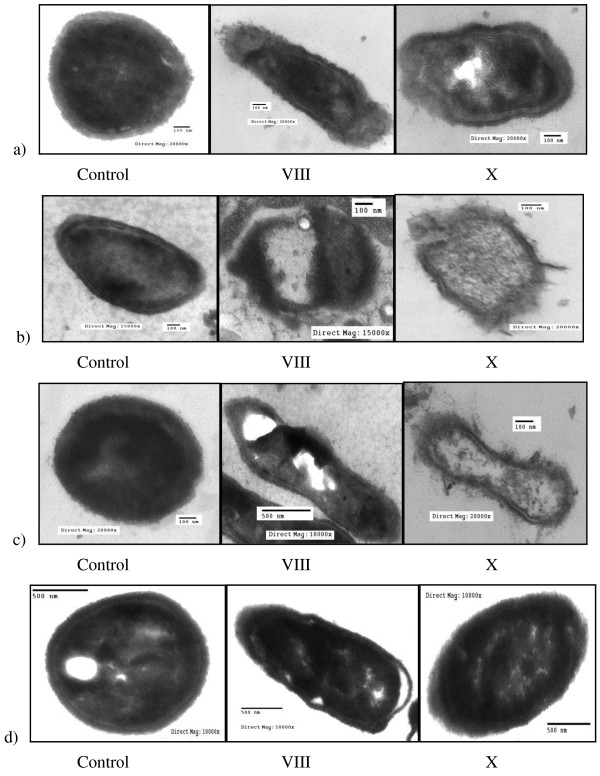
**Transmission electron micrograph of antimicrobial effects of polymers (VIII and X) against Gram-negative bacteria: (a) *Salmonella typhi*; (b) *Escherichia coli*; (c) *Pseudomonas aeruginosa*; and (d) fungus: *Candida albicans;* Effect of antimicrobial agent.**

**Figure 11:**
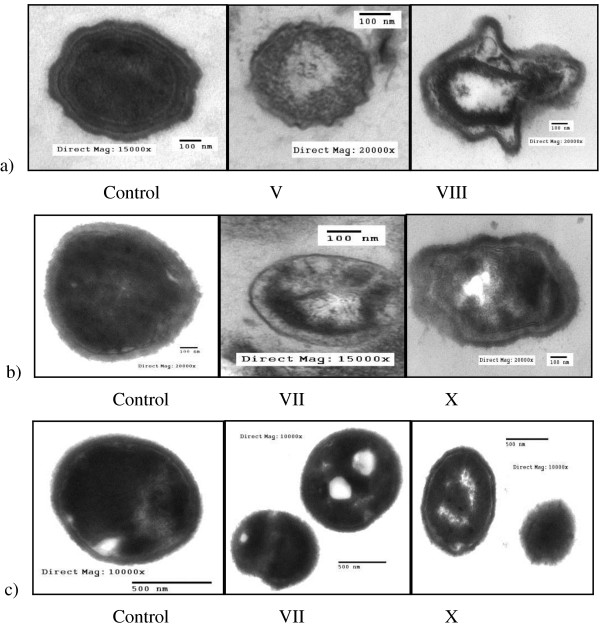
**Transmission electron micrograph of antimicrobial effects of polymers (V-VIII and X) against (a) Gram-positive bacteria: *Staphylococcus aureus;*(b) Gram-negative bacteria: *Salmonella typhi*and (c) fungi: *Aspergillus flavus*; Effect of spacer.**

Figures 10 &11 showed the TEM images of antimicrobial effects of the biocidal polymers (V-X) against fungi and pathogenic bacteria. Generally, the treated cells showed totally deformation and exhibited severe destruction. In contrast, the intact cells had a smooth surface with overall intact morphology.

Destructive effects of inhibitory agents against different microorganisms appear greatly causing firstly the rupture of cell wall that lead to the leakage of cytoplasmic contents, and then an observable shrinking of cytoplasmic size will appear. Deeper effects may appear as the coagulation of cell proteins, and precipitation of mineral crystals due to dehydration of cytoplasm. Finally, complete discharge of cytoplasmic contents will occur. Great effects can be easily observed with polymers (VIII & X) while lower effects were recorded for the other tested polymers [31].

The observed results from TEM images for the effect of the treated cells were confirmed by the estimation of both total soluble cell proteins concentration (Table 6) and the total cell ionic content (Table 7).

**Table 6:** Total cell protein concentration (mg/mL)

**Tested microorganism**	**Polymer code**						**Control**
	**V**	**VI**	**VII**	**VIII**	**IX**	**X**	
*Aspergillus flavus*	-*	-	17	-	-	15	31
*Aspergillus niger*	16	19	-	16	-	-	36
*Cryptpcoccus neoformans*	-	-	-	-	15	-	33
*Staphylococcus aureus*	14	-	-	18	16	15	35
*Pseudomonas aeruginosa*	-	20	-	-	-	-	32
*Salmonella typhi*	-	-	17	-	-	-	34

* Polymer not Applicable.

**Table 7:** Total soluble cell ions concentration (mol. = M)

**Tested microorganism**	**Polymer code**						**Control**
	**V**	**VI**	**VII**	**VIII**	**IX**	**X**	
*Aspergillus flavus*	-*	-	0.039	-	-	0.031	0.076
*Aspergillus niger*	0.042	0.047	-	0.035	-	-	0.073
*Cryptpcoccus neoformans*	-	-	-	-	0.029	-	0.073
*Staphylococcus aureus*	0.051	-	-	0.032	0.031	0.028	0.072
*Pseudomonas aeruginosa*	-	0.049	-	-	-	-	0.075
*Salmonella typhi*	-	-	0.037	-	-	-	0.074

* Polymer not Applicable.

### Estimation of total soluble cell proteins concentration

The more lowered protein content, the more effective the polymer in decreasing cellular activity. As shown in (Table 6), the total soluble proteins concentration was decreased to almost 50% or less compared to the control. Polymer (X) gave the highest inhibition of cell protein synthesis, and the lowest effect was recorded for polymer (VI).

### Estimation of total cell ionic content

In a similar way, the more lowered ion content, the more effective the polymer in leakage of cytoplasmic contents. Polymer (X) gave the highest inhibition of cell protein synthesis, and the lowest effect was recorded for polymers (V & VI) as shown in (Table 7).

## Conclusions

In our study; (1) Amine-terminated polyacrylonitriles were used as novel polymeric carriers for benzaldehyde derivatives as antimicrobial agents; (2) The antimicrobial screening results relieved the capability of polymers (V-X) to inhibit the growth of microorganisms and the inhibition zone diameter varied according to the tested microorganism as well as the polymer microstructure; (3) The inhibition zone diameter increased from polymer (VIII) passed to polymer (X) due to the increase in the number of phenolic hydroxyl group of the bioactive group; (4) The minimum inhibitory concentration (MIC) was recorded at 25 mg/ml for polymers (V & VIII) and decreased to 12.5 mg/ml for polymers (VII–X). Generally, the polymer samples killed 70–75% at MIC concentration the inhibitory effect varied according to the polymer microstructure and the organisms and (5) The TEM images showed that the treated cells were totally deformed and exhibited severe destruction. In contrast, the intact cells had a smooth surface with overall intact morphology.

Finally, it is anticipated that the prepared antimicrobial polymers would be of great help in the field of biomedical applications and biological water treatment.

## Competing interests

The authors declare that they have no competing interests.

## Authors’ contributions

ABA carried out all the experimental work. MHE and SSE designed the proposed methods and analyzed the data statistically together. All authors read and approved the final manuscript.
